# Hepatitis E virus chronic infection of swine co-infected with Porcine Reproductive and Respiratory Syndrome Virus

**DOI:** 10.1186/s13567-015-0207-y

**Published:** 2015-06-06

**Authors:** Morgane Salines, Elodie Barnaud, Mathieu Andraud, Florent Eono, Patricia Renson, Olivier Bourry, Nicole Pavio, Nicolas Rose

**Affiliations:** ANSES, Laboratoire de Ploufragan-Plouzané, BP 53, 22440 Ploufragan, France; Université européenne de Bretagne, 35000 Rennes, France; UMR 1161 Virology, ANSES, Laboratoire de Santé Animale, 94706 Maisons-Alfort, France; UMR 1161 Virology, INRA, 94706 Maisons-Alfort, France; UMR 1161 Virology, Paris Est University, École Nationale Vétérinaire d’Alfort, 94706 Maisons-Alfort, France

## Abstract

In developed countries, most of hepatitis E human cases are of zoonotic origin. Swine is a major hepatitis E virus (HEV) reservoir and foodborne transmissions after pork product consumption have been described. The risk for HEV-containing pig livers at slaughter time is related to the age at infection and to the virus shedding duration. Porcine Reproductive and Respiratory Syndrome Virus (PRRSV) is a virus that impairs the immune response; it is highly prevalent in pig production areas and suspected to influence HEV infection dynamics. The impact of PRRSV on the features of HEV infections was studied through an experimental HEV/PRRSV co-infection of specific-pathogen-free (SPF) pigs. The follow-up of the co-infected animals showed that HEV shedding was delayed by a factor of 1.9 in co-infected pigs compared to HEV-only infected pigs and specific immune response was delayed by a factor of 1.6. HEV shedding was significantly increased with co-infection and dramatically extended (48.6 versus 9.7 days for HEV only). The long-term HEV shedding was significantly correlated with the delayed humoral response in co-infected pigs. Direct transmission rate was estimated to be 4.7 times higher in case of co-infection than in HEV only infected pigs (0.70 and 0.15 per day respectively). HEV infection susceptibility was increased by a factor of 3.3, showing the major impact of PRRSV infection on HEV dynamics. Finally, HEV/PRRSV co-infection – frequently observed in pig herds – may lead to chronic HEV infection which may dramatically increase the risk of pig livers containing HEV at slaughter time.

**Electronic supplementary material:**

The online version of this article (doi:10.1186/s13567-015-0207-y) contains supplementary material, which is available to authorized users.

## Introduction

Hepatitis E virus is a non-enveloped single-stranded RNA virus causing an acute hepatitis in humans. It is mainly transmitted by the oro-fecal route and is responsible for clinical signs similar to hepatitis A virus infection [1]. Chronic cases have been described, mainly in immunocompromised patients [2,3]. Four HEV genotypes have been described. Genotypes 1 and 2 infect only humans and circulate in Asia, Africa and Central America in epidemic waves linked to the consumption of contaminated water [4–6]. Genotypes 3 and 4 are shared between humans and other animal species and are responsible for autochthonous sporadic cases in industrialized countries. In particular, the number of hepatitis E cases linked to genotype 3 has considerably increased in the last decade [6,7], in relation to better diagnosis. This genotype is highly prevalent in the swine population [8]. Some studies have shown that swine and human HEV strains are genetically very close [9] and HEV cross-species transmission has been proven [10,11]. Moreover, a number of autochthonous cases have been related to the consumption of undercooked pork meat, especially liver products [12–16]. Thus, hepatitis E is now recognized as a foodborne zoonosis for which domestic pigs are considered as the main reservoir in developed countries [7,17,18]. Understanding factors influencing the transmission dynamics of HEV in pig herds is crucial to limit the risk of an introduction of contaminated products in the food chain. Several studies have described experimental HEV infection trials via oral or intravenous route [19–24] but few studies were aimed at quantifying HEV transmission [20,25]. The results of these studies on HEV transmission were different than those observed in pig farms on the field, with the latent and infectious period estimates being generally longer than in experimental trials [26–28]. Moreover, a high variability of HEV infection dynamics is observed on pig farms and has not yet been fully explained [29]. Some factors affecting swine immune response may also influence the course of HEV infection. Porcine Respiratory and Reproductive Syndrome Virus (PRRSV) is a highly prevalent virus that impairs the immune response. It has been detected together with HEV in several studies but no evidence of a causal relationship has been shown to date [30–32]. Since chronic cases in humans are generally linked to immunosuppressive conditions [33–36], PRRSV might be suspected as a frequent co-factor affecting the features of HEV infection in pigs.

The impact of a PRRSV infection on HEV infection dynamics (in terms of viral shedding duration and quantity, transmission and humoral immune response) has therefore been studied through a transmission experiment involving HEV/PRRSV co-infection of specific-pathogen-free (SPF) pigs compared to an infection trial with HEV only that was previously led in our facilities, under the same conditions [25].

## Materials and methods

### HEV-only infection experiment

A transmission trial with HEV only has been carried out before the co-infection experiment [25]. The experiment was conducted in Anses air-filtered level-3 biosecurity facilities. Briefly, sixty-eight SPF Large-White piglets were used for the experiment. Eight pigs were kept as negative controls and the others were allocated to six rooms containing two pens per room. Rooms 1 to 3 were used to evaluate direct and environmental transmission, whereas Rooms 4 to 6 were used to examine between-pen transmission. The inoculated pigs received orally 10^8^ ge (genome equivalent) under a volume of 10 mL of a genotype 3 HEV suspension (strain FR-SHEV3e, Genbank access number JQ953665). Individual fecal samples were collected four days before inoculation and three times per week from 0 to 39 days post-infection (dpi) when the pigs were killed for necropsy. Blood samples were collected twice a week during the same period and clinical signs and rectal temperature were monitored on a daily basis.

### HEV/PPRSV co-infection experiment

#### Animal housing conditions and inoculation

**Figure 1:**
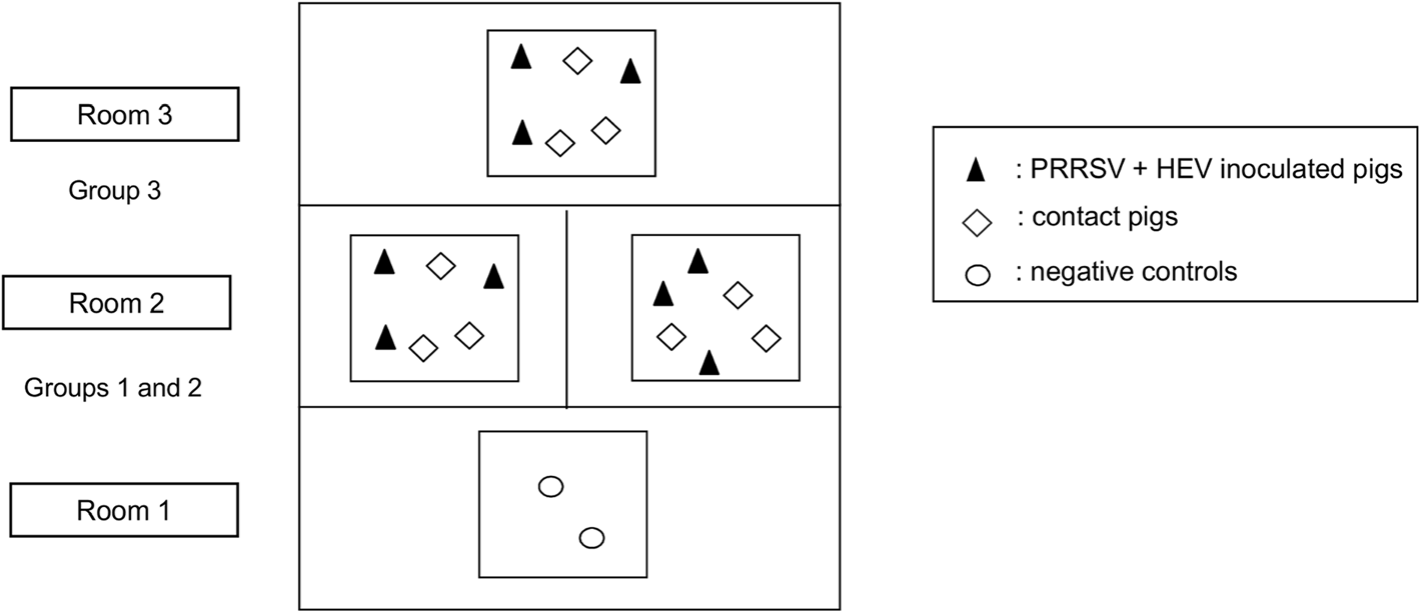
**Experimental design of the co-infection experiment.** Inoculated and susceptible contact animals are represented by black triangles and white diamonds, respectively. Rooms 2 and 3 contained three pens housing three HEV/PRRSV co-inoculated (black triangles) and three susceptible contact pigs (white diamonds). One negative control group was housed in Room 1.

The experiment was conducted in the same Anses air-filtered level-3 biosecurity facilities. Twenty five-week-old SPF Large-White piglets were included in the study; they were HEV and PRRSV free and they did not have any maternal antibodies against these two viruses. Pigs were housed in metallic flat decks with a punched floor for feces and urine evacuation. As in the field situation, fecal material could accumulate in the corners and was not removed during the trial. Three rooms were used: two negative control pigs were housed in Room 1 whereas the 18 remaining piglets were randomly allocated to 3 independent pens distributed in Room 2 and Room 3 (6 piglets per pen) stratifying on gender (3 males and 3 females per pen), weight and the litter they came from. Room 2 contained 2 pens separated by a solid partition to prevent contamination of a pen by the other one (Figure 1). The average weights at weaning (sd) were 9.5 kg (2.7), 9.3 kg (1.6), 9.3 kg (2.3) and 9.3 kg (1.4) for Controls and groups #1, #2 and #3 respectively. In each pen, 3 piglets were inoculated with both HEV and PRRSV at day 0. For inoculation, piglets to be inoculated were grouped in a pen and they were put in contact with their corresponding pen-mates at day 1. The 3 inoculated piglets received the following: (i) orally 10^8^ ge under a volume of 10 mL of a genotype 3 HEV suspension (strain FR-SHEV3e, Genbank access number JQ953665) prepared according to the protocol previously described in Andraud et al. [25] (ii) and by nasal route 2.5 mL per nostril of a PRRSV suspension (strain PRRS-2005-29-24-1 “Finistere”, genotype 1, subtype 1) titrating 10^5^ TCID_50_/mL. The experiment was performed in accordance with EU and French regulations on animal welfare in experiments. The protocol was approved by the Anses/ENVA/UPEC ethical committee (agreement #16 with the National committee for Ethics in animal experimentation).

#### Data collection

Individual fecal samples were collected three days before inoculation and three times a week until the end of the experiment (49 dpi). Blood samples were collected before inoculation and once a week until the end of the experiment. Clinical examination was also performed (clinical signs, rectal temperature, feces consistence, weight, food consumption and trough cleanliness were recorded daily). Euthanasia was carried out by intravenous injection of 1 g/50 kg live weight of Nesdonal® (thiopental-sodium, Merial, Lyon, France) followed by exsanguination. Necropsy was performed and liver samples were taken.

Because HEV is a zoonotic agent, strict biosecurity measures were applied to prevent any transmission from pigs to animal technicians.

#### Virology and serology analyses

HEV RNA quantification in fecal and liver samples was performed, after manual total RNA extraction, using real-time quantitative RT-PCR as described in Barnaud et al. [37] and Andraud et al. [25]. The results were expressed in terms of Cycle threshold (Ct). Standard quantification curves were produced by plotting the Ct values against the logarithm of the input copy numbers of standard RNA. Standard RNA was obtained after in vitro transcription of a plasmid pCDNA 3.1 ORF 2–3 HEV, as described in Barnaud et al. [37]. The results are expressed in genomic copy number per gram of feces (ge/g).

The detection of anti-HEV antibodies was performed using the HEV ELISA 4.0v kit (MP Diagnostics, Illkirch, France) according to the manufacturer’s instructions, except the serum quantity used (10 μL instead of 20 μL). This sandwich ELISA allows the detection of all antibody classes (IgG, IgM and IgA) and uses a recombinant antigen that is present in all HEV strains. Samples were positive when the optical density at 450 nm wavelength obtained for the sample was higher than the threshold defined as the mean for negative controls + 0.3.

PRRSV RNA detection in sera was performed using a real-time RT-PCR as described in Charpin et al. [38]. Briefly, RNA extraction was performed using the NucleoSpin® 8 virus kit (Macherey-Nagel, Düren, Germany) according to the manufacturer’s instructions. RNA detection was then performed using the mix GoTaq 1-Step RT-qPCR System (Promega) supplemented with probes and specific primers of the target gene (ORF7 pan-PRRSV) and of the internal reference gene (swine Beta-Actin). The RT-PCR was performed on a Bio-Rad Chromo4 real-time PCR detection system (Bio-Rad) according to the following program: 50 °C for 30 min, 94 °C for 2 min followed by 45 cycles of 94 °C for 15 s and 60 °C for 30 s. The results are expressed in Ct.

### Models

#### Estimation of durations related to HEV infection dynamics

The latent and infectious period durations and the duration of the period required to produce anti-HEV antibodies were estimated using survival data analyses. For each inoculated animal, the latent period was determined as the time elapsed between the inoculation day and the date of the first positive fecal sample for HEV RNA. The latent period after inoculation was fitted to a gamma distribution, from which the shape and scale parameters were estimated by the maximum log-likelihood method. A nonparametric bootstrap procedure was used to determine the 95% confidence interval of the parameter estimates.

A parametric model for survival data was built to estimate the duration of the infectious period, using the RT-PCR performed on livers after euthanasia as the last observation date. Two parametric models were tested (log-normal and Weibull distributions of survival times) and compared using the Akaike Information Criterion (AIC).

The impact of PRRSV co-infection on the time to HEV seroconversion was also studied with a parametric survival model applied to the data from the co-infection trial and the only HEV infection experiment [25]. The link between the earliness of the HEV antibody response and the duration of the infectious period was studied with a Cox model. The immune response was considered as absent or late if the delay before seroconversion was longer than 25 dpi, and as early if it was shorter than 25 dpi [39].

All analyses were performed using the R software (survreg and coxph functions) [40].

#### Quantification of HEV shedding, environmental accumulation and transmission

The distributions of HEV shed viral loads with time (with and without co-infection) are represented with box plot series. A linear mixed model (proc Mixed, SAS 9.3, [41]) which took into account repeated measurements with time was built to study the difference in the quantity of HEV shed particles between co-infected and non co-infected pigs.

The environmental load corresponds to the accumulation of viral particles in the environment through fecal shedding by infected animals, which is partially compensated by the clearance rate hereafter denoted *δ*. The clearance rate takes into account feces elimination through the metallic flat deck and HEV intrinsic mortality in the environment. As described in Andraud et al. [25], for each pen (*k*) and every sampling time (*t*_*i*_), the average quantity of genome equivalent shed in the environment per gram of feces was calculated with:

$$ {V}_k\left({t}_i\right)={\displaystyle {\sum}_j{V}_k^j\left({t}_i\right)/{N}_k} $$

where *V*_*k*_^*j*^(*t*_*i*_) represents the quantity of virus shed per gram of feces in pen *k* by pig *j* at time *t*_*i*_ and *N*_*k*_ the total number of animals in pen *k*. Thus the cumulated viral load in the environment of pen *k* between two sampling times *t*_*i*_ and *t*_*i* + 1_ is given by the equation:

$$ {E}_{ki}={E}_k\left({t}_{i+1}\right)=\left({E}_k\left({t}_i\right)+{\displaystyle {\displaystyle {\int}_0^{\varDelta t}{V}_k\left({t}_i+u\right){e}^{\delta u}du}}\right){e}^{-\delta \varDelta t},\;\mathrm{with}\;\varDelta t={t}_{i+1}-{t}_i. $$

Two HEV transmission routes were investigated in this study: (i) transmission due to direct contact between infected and naïve pigs; (ii) indirect transmission via an environmental reservoir of the virus in the pen. A Bayesian model similar to the one described in Andraud et al. [25] was used. Briefly, on each sampling interval *D*_*i*_ = [*t*_*i*_, *t*_*i* + 1_] of duration *d*_*i*_, the probability for a susceptible pig *j* housed in pen *k* to escape infection is given by:

$$ {p}_i^{(k)}= exp\left(-{d}_i\left({\beta}_w{\pi}_i^{(k)}+{\beta}_E^{(w)}\frac{E_{ki}^{(w)}}{N}\right)\right), $$

where *π*_*i*_^(*k*)^ represents the proportion of shedding pigs in the time interval *D*_*i*_ located in pen *k*, *E*_*ki*_^(*w*)^ is the environmental pool of viral particles in time interval *D*_*i*_ in the pen, *β*_*w*_ is the within-pen transmission rate by direct contact and *β*_*E*_^(*w*)^ is the within-pen environmental transmission rate. For each pig *j*, the time interval in which the infection occurred was determined by estimating the latent period *λ*_*j*_. Let ${D}_{I_j}=\left[{t}_{I_j},\ {t}_{I_j+1}\right]$ denote the time interval during which the first positive fecal sample was detected in pig *j*. The contribution of contact animal *j* in pen *k* to the likelihood model, i.e. the probability for its first positive fecal sample to stand in the interval ${D}_{I_j}=\left[{t}_{I_j},\ {t}_{I_j+1}\right]$ is:

$$ {L}^{(j)}\left({D}_I,{\pi}_w,E\Big|{\beta}_w,{\beta}_E^{(w)},\lambda, \delta \right)=\left\{{\displaystyle \prod_{i=1}^{I_j}}{p}_{l-1}^{(k)}\ \left(1-{p}_{I_j}^k\right)\right\}\times {f}_{Lat}\left({\lambda}_j,\ \alpha, s\right), $$

The probability of infection (given by the first term of the equation aforementioned) is weighted by the probability that the estimated latent period *λ*_*j*_ is consistent with the data observed in inoculated animals. *f*_*Lat*_ represents the prior distribution of the latent period based on the estimation of the latent period in inoculated animals. The global likelihood is given by:

$$ L\left({D}_I,{\pi}_w,E\Big|{\beta}_w,{\beta}_E^{(w)},\lambda, \delta \right)={\displaystyle \prod_{j=1}^{N_c}}{L}^{(j)}\left({D}_I,{\pi}_w,E\Big|{\beta}_w,{\beta}_E^{(w)},\lambda, \delta \right), $$

where *N*_*c*_ is the total number of contact pigs.

The direct and indirect transmission rates *β*_*w*_ and *β*_*E*_^(*w*)^ respectively, the latent period *λ*_*j*_ for each contact animal and the HEV clearance rate were estimated by Bayesian inference using Monte Carlov Markov Chain. An informative prior distribution based on Andraud et al. [25] was used for the environmental clearance rate *δ*, which was assumed to be normally distributed with mean 0.3 and standard deviation 0.075. The prior distributions of transmission parameters were based on the results obtained by Andraud et al. [25]; they were constructed such that the expected value is equal to the posterior mean and 33% of the prior mass covers the 95% confidence interval for parameters derived from data obtained by Andraud et al. [25,42] (normal distribution (−2,3) and (−13.5,5) for *β*_*w*_ and *β*_*E*_^(*w*)^ respectively). The prior distribution of the latent period in contact pigs was based on the distribution of the latent period in inoculated pigs (gamma distribution Γ(26,2)).

Parameter updating was performed sequentially by the Metropolis-Hastings algorithm. Three chains were run with random initial conditions, 110 000 steps per chain, a burnin of 10 000 steps and thinning parameter of 10. Convergence was assessed by visual inspection and diagnostic tests (autocorrelation, Heidelberger, Gelman-Rubin diagnostics).

The whole model was performed using the R software [40].

## Results

### HEV-only infection experiment

In this trial, the average HEV latent period in inoculated animals lasted 6.9 days (5.8; 7.9) and average infectious period lasted 9.7 days (8.2; 11.2) (Table 1) [25]. Direct transmission rate was estimated at 0.15 (0.03; 0.31) pigs per day and indirect transmission rate was estimated at 2·10^−6^ g/ge/day (1·10^−7^; 7·10^−6^) (Table 1) [25]. HEV serology results on individual blood samples for HEV-only infected pigs are presented in Additional file 1 [25].

**Table 1:** Summary of the infectious dynamics parameters and comparison with data from the HEV-only infection experiment [25]

	HEV + PRRSV	HEV alone [25]
Latent period (days)	13.4	7.1
	(8.6; 17.1)	(3.2; 12.3)
Infectious period (days)	48.6	9.7
	(27.9; 84.6)	(8.2; 11.2)
Seroconversion period (days)	43.1	26.3
	(35.7; 52.2)	(23.5; 29.5)
Direct transmission (days^−1^) *β*_*w*_	0.70	0.15
	(1.2^.^10^−3^; 3.67)	(0.03; 0.31)
Indirect transmission (g/ge/d) *β*_*E*_^*w*^	6.6^.^10^−6^	2.0^.^10^−6^
	(1.4^.^10^−10^; 1.3^.^10^−4^)	(1.1^.^10^−7^; 7.0^.^10^−6^)

*β*_*w*_ is the direct transmission rate, defined as the mean number of newly infected pigs generated by a single infectious individual in a fully susceptible population per day. *β*_*E*_^*w*^ represents the within-pen transmission rates related to the environmental component, defined as the mean number of newly infected pigs per HEV genome equivalent per gram of feces in the environment (see text for more details). Numbers in brackets are the upper and lower limits of the 95% credibility interval.

### HEV shedding and seroconversion in the context of HEV/PRRSV co-infection

**Figure 2:**
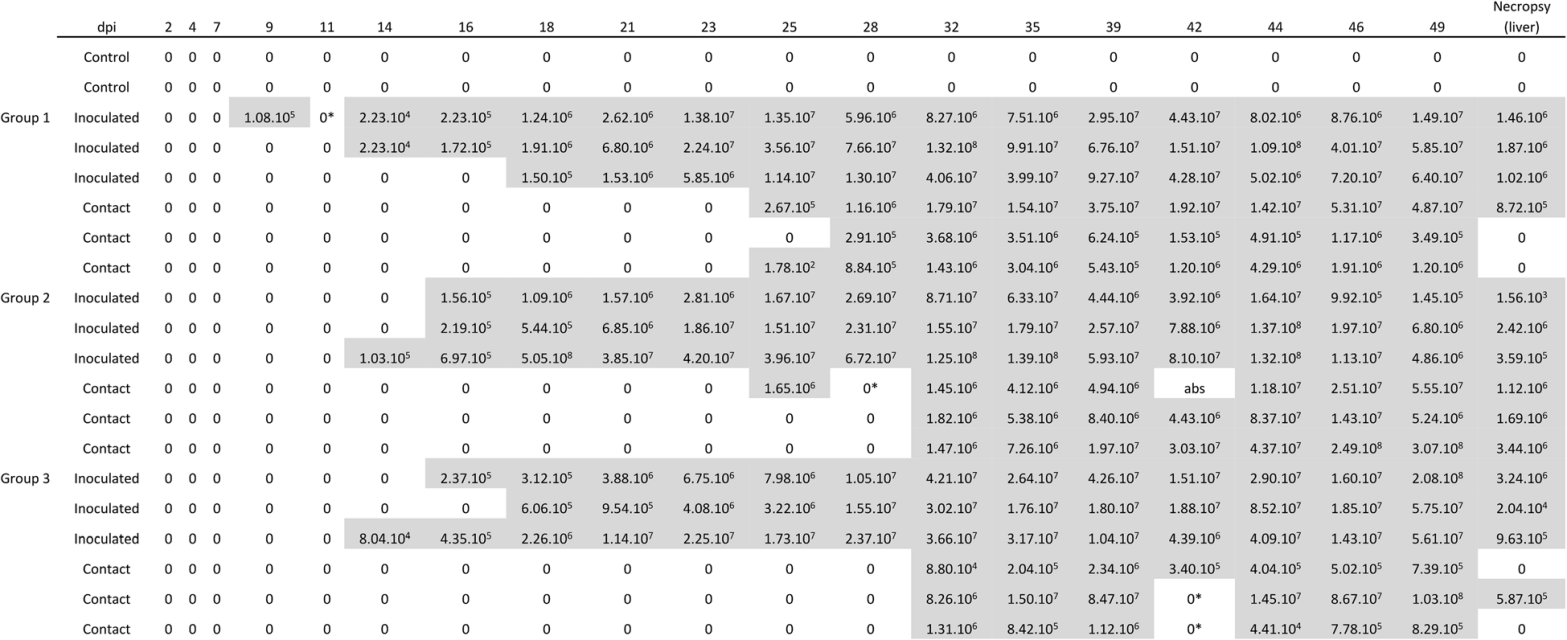
**HEV RNA quantification in fecal and liver samples from HEV/PRRSV co-infected animals and contact pigs.** Quantitative HEV RT-PCR results on individual fecal samples (HEV copies/g of feces) at each sampling time and from liver samples at necropsy. Shaded zones correspond to periods in which infected individuals were considered infectious, corresponding to the time between the first and last HEV positive fecal samples for each animal. dpi: day post infection; *tested in duplicate; abs: missing.

**Figure 3:**
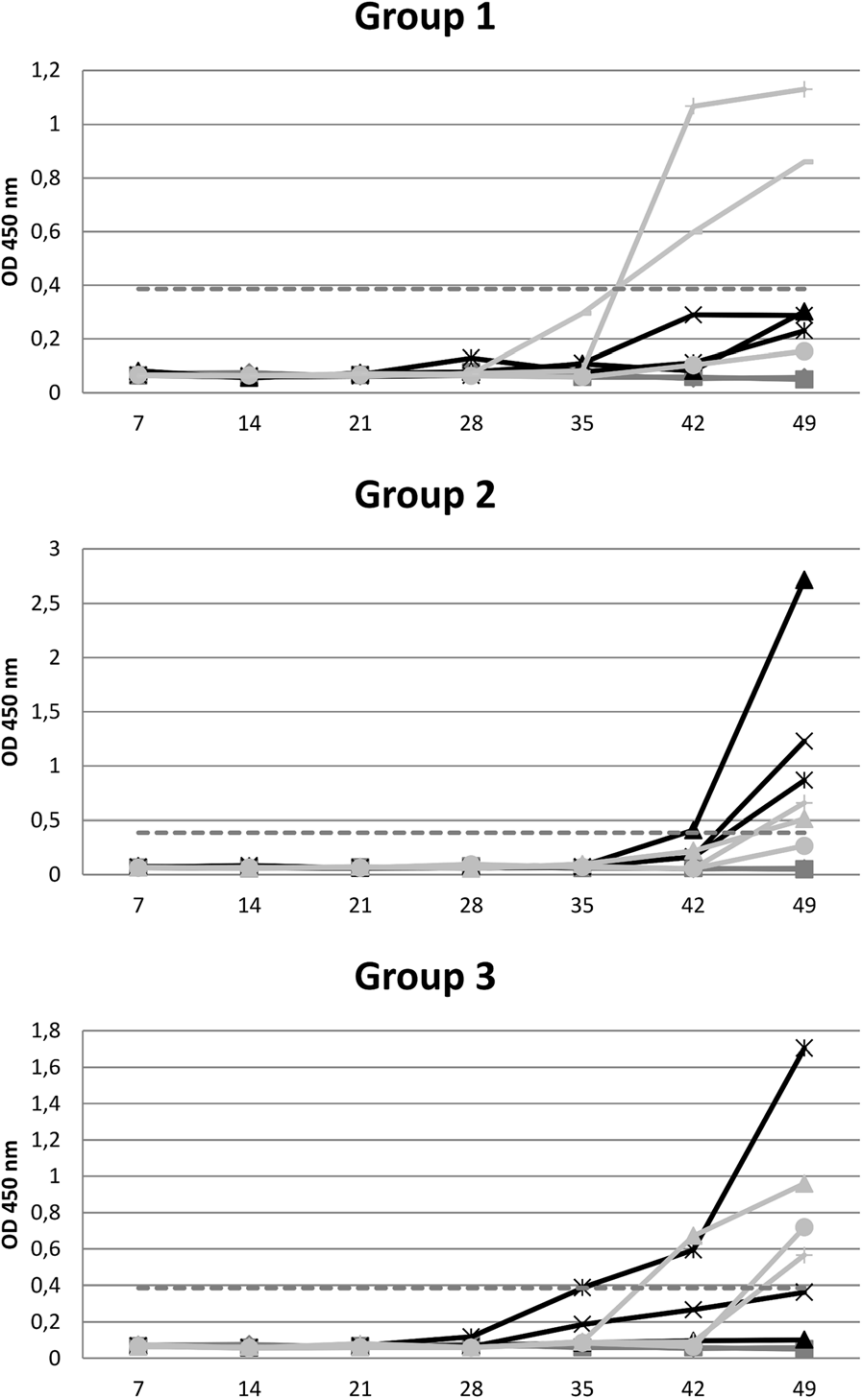
**HEV serology results on individual sera samples from HEV/PRRSV co-infected animals and contact pigs.** Optical density (450 nm) values of ELISA test HEV 0.4v per animal at different days post infection. For each group, inoculated animals are indicated in black (*n* = 3), contact pigs in light grey (*n* = 3) and negative control in dark grey (*n* = 2). The cut off value is indicated by a dashed grey line.

HEV infection data are presented in Figures 2 and 3 for quantitative RT-PCR on fecal samples and serological results respectively. In our trial, all inoculated animals were infected by HEV. None of the 2 negative-control pigs excreted HEV from day 3 to day 49. Inoculated and contact animals started to shed HEV between 9 and 18 dpi and between 25 and 32 dpi respectively. All exposed individuals shed HEV until the end of the trial (49 dpi) (Figure 2). At the necropsy stage, 14 livers out of 18 were positive, the 4 negative livers being from contact pigs (Figure 2).

The detection of anti-HEV antibodies was performed on all groups of animals until 49 dpi (Figure 3). None of the negative controls showed anti-HEV antibody response. Only 4 inoculated animals out of 9 produced anti-HEV antibodies between 35 and 49 dpi, 3 in group 2 and one in group 3; none of the inoculated animals from group 1 seroconverted. Seven contact individuals out of 9 seroconverted between 42 and 49 dpi, two from groups 1 and 2 and all three contact animals from group 3 (Figure 3).

### PRRSV infection and seroconversion in the context of HEV/PRRSV co-infection

**Figure 4:**
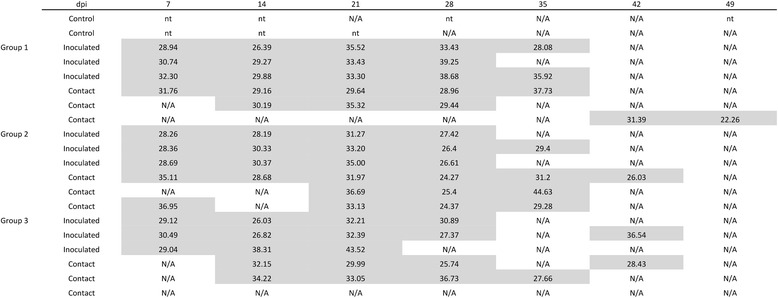
**PRRSV RT-PCR results on individual blood samples.** Shaded zones correspond to periods in which individuals were considered viremic. The results are expressed in terms of Ct. dpi: day post infection; nt: not tested; N/A: not amplified; Ct: cycle threshold.

All animals inoculated with PRRSV were viremic from the first sampling time (7 dpi). The viremia of contact animals started between 7 and 42 dpi. One contact individual did not show any detectable PRRSV viremia during the experiment (Figure 4). Finally, all animals except 2 contact individuals were viremic for PRRSV before HEV shedding was detected.

Regarding clinical data (data not shown), inoculated and contact animals showed hyperthermia (rectal temperature >40 °C) between 1 and 14 dpi and 14 and 28 dpi, respectively. Co-infected pigs necropsied at 49 dpi did not show any macroscopic lesion possibly linked to hepatitis.

### Quantification of HEV infection dynamics parameters in the context of HEV/PRRSV co-infection

Convergence of MCMC was assessed through visual inspection and conventional diagnostic tests. Heidelberger and Geweke diagnostics failed to reject the convergence hypothesis, which was also supported by the Gelman-Rubin test based on three independent chains with a potential scale reduction factor (PSRF) close to 1.0 (≤1.02) (Additional file 2).

#### HEV latent and infectious periods

The duration of the latent period in pigs inoculated with HEV and PRRSV was fitted to a gamma distribution with shape parameter *α* = 25.7 (11.6; 180.4) and scale parameter *s* = 0.5 (0.08; 1.1) leading to an estimated mean duration of the latent period of 12.9 days (12.8; 14.4). In contact animals, individual distributions of latent periods (Additional file 2) were merged to obtain a global distribution of the latent period, leading to a mean latent period duration of 13.4 days (8.6; 17.1) (Table 1).

The duration of the infectious period was fitted to a log-normal distribution, leading to an estimated mean duration of the infectious period of 48.6 days (27.9; 84.6) (Table 1).

#### Estimation of time to HEV seroconversion

**Figure 5:**
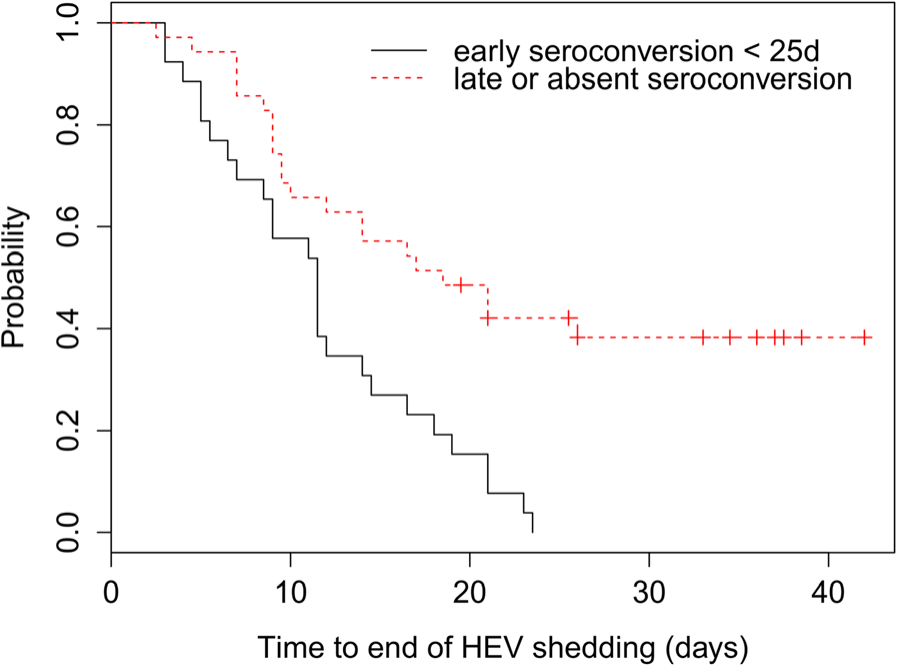
**Survival curves of time-to end of HEV shedding according to early or late HEV seroconversion.** The black and red survival curves correspond to the duration of the infectious period in pigs having an early seroconversion (less than 25 dpi) or a late or absent seroconversion (more than 25 dpi) respectively.

Time-to HEV seroconversion was fitted to log-normal distribution, with means 43.1 days (35.7; 52.2) with PRRSV co-infection and 26.3 days (23.5; 29.5) with only HEV infection (Table 1). The duration of the infectious period was significantly associated with the earliness of the humoral immune response. An absent or late immune response was related to a lengthening of the infectious period duration showed by a delay in time-to end of shedding (Hazard Ratio HR = 0.35 (0.19; 0.64)) (Figure 5).

#### HEV shedding and accumulation in the environment

**Figure 6:**
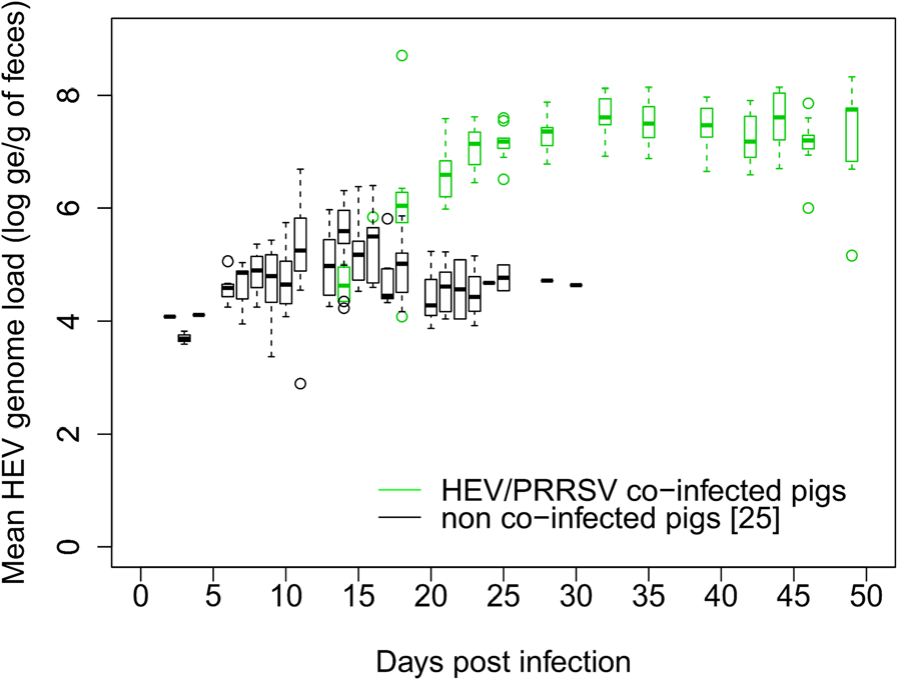
**Distribution of the number of HEV genome equivalent (log ge/g feces) shed by individual pigs with time in inoculated animals with or without PRRSV co-infection.** Co-infected animals are indicated in green (*n* = 9), only-HEV infected animals [25] are in black (*n* = 18).

The distribution of the HEV shed viral load with time (with and without co-infection) is shown in Figure 6. PRRSV infection was found to be significantly associated with the increase of the quantity of HEV particles shed by inoculated animals (*P =* 0.05) from the linear mixed model accounting for repeated measurements. The interaction between time and PRRSV infection was also significant and positive, i.e. the impact of the PRRSV infection increased with time (*P =* 0.04). However, the effect of the PRRSV infection was not found to be statistically significant in contact animals (*P >* 0.05).

**Figure 7:**
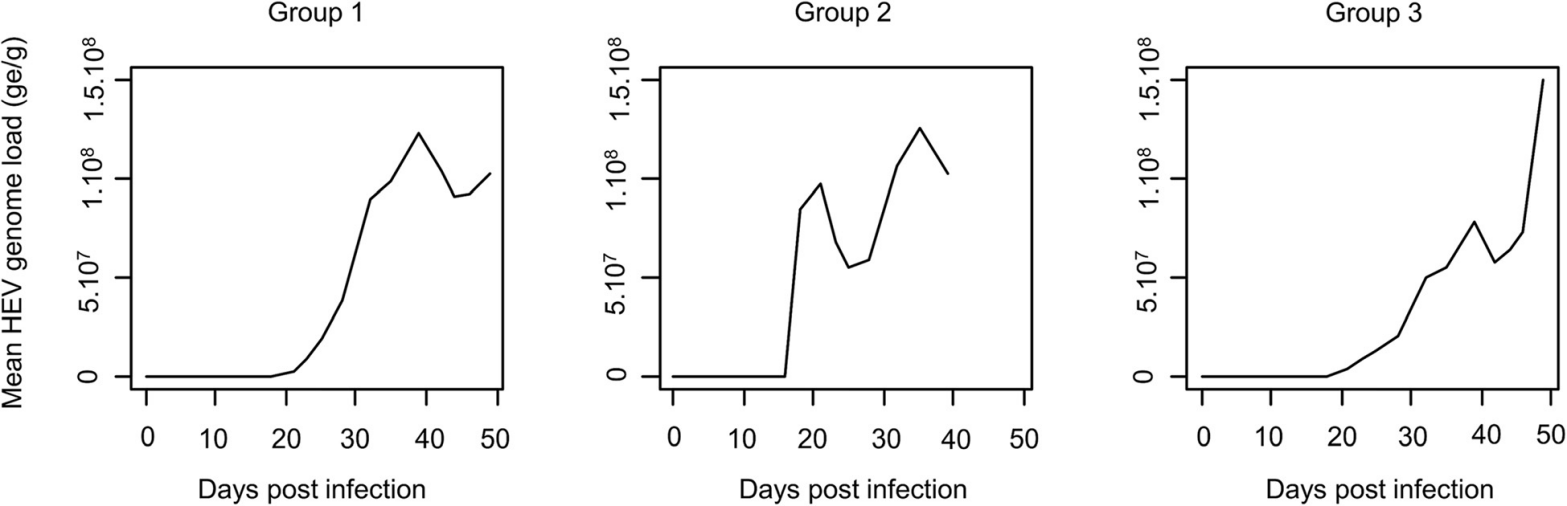
**Estimation of HEV environmental accumulation with PRRSV/HEV co-infection.** Evolution of the estimated HEV genome load (ge/g) in the environment of each pig group of the PRRSV/HEV co-infection experiment.

The viral load accumulated in the environment was modeled for each experimental pen (Figure 7). The environment was HEV-free until 15 to 20 dpi; then the environmental load increased and reached 1.0.10^8^ to 1.5·10^8^ ge/g of feces until the end of the trial.

#### HEV transmission parameters

The results show that, in experimental conditions, one infectious pig was able to infect 0.70 pig per day by direct contact (*β*_*w*_ = 0.70 (1.18·10^−3^; 3.67)) (Table 1). The indirect transmission rate can be considered as the average number of animals that can be infected by a single genome equivalent present in the pen environment (*β*_*E*_^(*w*)^ = 6.59·10^−6^ g/ge/day (1.43·10^−10^; 1.27·10^−4^)). In other words, the inverse of *β*_*E*_^(*w*)^ corresponds to the average number of viral copy number of genome per gram of feces in the environmental pool required to infect one animal in one day, i.e. 1.51·10^5^ ge/g/day (7.86·10^3^; 7.00·10^9^) (Table 1).

## Discussion

Several studies suggested a possible link between HEV and PRRSV infections [30–32]. Our study was aimed at evaluating the impact of PRRSV infection on hepatitis E dynamics of infection through an experimental HEV/PRRSV co-infection trial. As shown in Table 1, the comparison of the results with those derived from a previous infection trial with HEV alone [25] evidenced a modification of hepatitis E infection dynamics in the presence of PRRSV. Although the two trials were not carried out simultaneously, they were conducted under the same experimental conditions making the comparison of the results fully relevant (same experimental facilities, same handlers, pigs from the same SPF herd and genetically similar, same age of the animals, same sex ratio, same HEV strain, same dose, same inoculation protocol and same contact structure).

HEV shedding was delayed in case of PRRSV co-infection, with a latent period estimated to 13.4 days, against 7.1 days with HEV alone [25], i.e. an increase by a factor of 1.9. In the Bouwknegt et al. trial, the latent period was estimated at only 3 days in intravenously inoculated animals [20], confirming that the route of inoculation modifies viral fate. The infectious period was longer with PRRSV co-infection: 48.6 days, against 9.7 days with HEV alone, i.e. an increase by a factor of 5 (*p* < 0.01). These results were therefore closer to estimates obtained from field data (27 days (20; 39)) than experimental results obtained with HEV only [26]). In the trial described by Bouwknegt et al., the infectious period was estimated between 13 and 49 days according to replications, showing a high inter-individual variability [20]. Moreover, the origin of the animals included in this study and their status regarding PRRSV were not mentioned.

HEV shedding in inoculated individuals was also significantly increased with PRRSV/HEV co-infection. However, the effect of PRRSV infection on the quantity of shed viral particles was not significant in contact animals. This could be explained by the low number of animals included – especially since one contact animals was lately infected by PRRSV and another did not show any PRRSV viremia during the experiment – and by a large inter-individual variability in contact animals. As a consequence of the longer shedding period and the higher quantity of viral particles shed in feces of co-infected animals, the viral load accumulated in the environment was higher with PRRSV co-infection with more than 10^8^ HEV ge/g of feces estimated in the environment, which causes a higher and longer infection pressure on susceptible animals. The direct transmission rate when animals were co-infected was increased by a factor of 4.7 (0.70 versus 0.15 per day with HEV infection only [25]). Thus the direct transmission route played a more important role in HEV transmission when animals were co-infected which was consistent with the larger amount of HEV particles shed individually than in HEV infected pigs only. The indirect transmission rate was 3.3 times higher with co-infection (6.6·10^−6^ and 2.0·10^−6^ g/ge/day respectively [25]). Otherwise stated, 3.3 times less viral particles were required to infect a co-infected animal (1.5·10^5^ versus 5.0·10^5^ ge/g for HEV only infected piglets [25]). Because inoculated and contact animals (except two contact pigs) were infected by PRRSV before HEV shedding, these data suggest a higher HEV susceptibility in PRRSV co-infected pigs. In a model built from an experimental HEV infection by intravenous route, Bouwknegt et al. showed that the HEV oral dose for which the infection probability was equal to 50% would be 1.4·10^6^ ge/g [22], which was 10 times more than the dose required to infect a PRRSV co-infected pig in our study. These data are consistent with the hypothesis of a higher HEV infection susceptibility in PRRSV co-infected pigs.

The time-to HEV seroconversion was 1.6 times longer in PRRSV co-infected pigs than in HEV only infected pigs (43.1 and 26.3 days respectively [25]). This impaired immune response was significantly associated with a lengthening of the infectious period duration and could thus explain the presence of viral particles in livers when pigs were euthanized more than 49 days post infection for the inoculated ones. However, this study did not aim at investigating the mechanisms leading to a possible immune failure linked to PRRSV infection and the mechanisms causing a chronic HEV infection. In humans, immunopathogenic mechanisms leading to chronic hepatitis E are poorly known. The role of cellular immunity in chronic hepatitis E control has been shown [3,35,36]. A study was led on patients suffering from HIV and chronically infected with HEV [34]. One of them had a low anti-HEV lymphocyte T CD4+ rate, a persistent viremia (longer than 24 months) and a delayed anti-HEV seroconversion. Thus, though immune mechanisms still need to be clarified, literature data suggest that an impaired innate and adaptive immune response could lead to chronic HEV infection in humans. In pigs, the immunopathogenic mechanisms linked to PRRSV infection are not fully understood yet, but PRRSV infection clearly results in a late adaptive immune response [43,44]. Thus the delayed anti-HEV seroconversion and the lengthening of the infectious period duration that we observed in PRRSV co-infected pigs seem consistent with the immunopathogenic mechanisms of chronic hepatitis E that have been described in humans (impaired cellular and humoral immune response) and could be explained by a specific orientation of the immune response linked to PRRSV infection. The increase of the duration of the latent period might be explained by the activation of the innate immune response linked to the PRRSV infection, delaying HEV shedding but this would require further work to assess the underlying mechanisms.

To our knowledge, this work is the first study focusing on the impact of HEV/PRRSV co-infection on hepatitis E epidemiology in pigs. These results show that PRRSV has a major impact on HEV infection dynamics and that HEV/PRRSV co-infection could lead to extended HEV shedding and maybe chronic infection. This chronicity may dramatically increase the risk of pig livers containing HEV at slaughter age. Immunopathogenic mechanisms leading to a chronic HEV infection have to be further investigated. This study shows an important interaction between an animal health concern - PRRSV, which dramatically affects the competitiveness of pig farms, and a zoonotic pathogen - HEV, which has a major impact in human health. These data emphasize the necessity to manage human and animal health globally and the importance of PRRSV eradication programs, which could be a major lever in the control of hepatitis E.

## Additional files

Additional file 1:
**HEV serology results on individual sera samples for only-HEV infected pigs [** 25 **].** Optical density (450 nm) values of ELISA test HEV 0.4v per animal at different day post infection. Shaded zones correspond to the period in which individuals were considered HEV seropositive. dpi: days post infection, abs: missing. Additional file 2:
**Estimation of transmission parameters by Bayesian inference (MCMC estimation, 3 chains, 110 000 iterations, 10 000 burnin iterations, thinning interval = 10).** *β*_*w*_ is the direct transmission rate, defined as the mean number of newly infected pigs generated by a single infectious individual in a fully susceptible population per day. *β*_*E*_^*w*^ represents the within-pen transmission rates related to the environmental component, defined as the mean number of newly infected pigs per viral particle per gram of feces in the environment. *δ* is the HEV clearance rate, taking into account feces elimination through the metallic flat deck and HEV destruction in the environment. *λ*_*1*_ to *λ*_*9*_ are latent periods for contact animals (see text for more details).
