# Supplementary material for: Hepatitis E virus chronic infection of swine co-infected with Porcine Reproductive and Respiratory Syndrome Virus
Source: Vet Res. 2015 Jun 6;46(1):55. doi: 10.1186/s13567-015-0207-y (PMC4456777; doi:10.1186/s13567-015-0207-y)
Supplement: Additional file 2: — Estimation of transmission parameters by Bayesian inference (MCMC estimation, 3 chains, 110 000 iterations, 10 000 burnin iterations, thinning interval = 10). β w is the direct transmission rate, defined as the mean number of newly infected pigs generated by a single infectious individual in a fully susceptible population per day. β Ew represents the within-pen transmission rates related to the environmental component, defined as the mean number of newly infected pigs per viral particle per gram of feces in the environment. δ is the HEV clearance rate, taking into account feces elimination through the metallic flat deck and HEV destruction in the environment. λ 1 to λ 9 are latent periods for contact animals (see text for more details). [file 13567_2015_207_MOESM2_ESM.docx]

| Parameter | Parameter estimates | | Gelman-Rubin test, potential scale reduction factor (PRSF) | |
| --- | --- | --- | --- | --- |
|  | **median** | **CI* 95%** | **median PRSF** | **CI 97,5%** |
| $\boldsymbol{\beta}_{\boldsymbol{w}}$ | 0.70 | (1.18.10^-3^;3.67) | 1.02 | 1.08 |
| $\boldsymbol{\beta}_{\boldsymbol{E}}^{\boldsymbol{w}}$ | 6.59.10^-6^ | (1.43.10^-10^;1.27.10^-4^) | 1.01 | 1.02 |
| *δ* | 0.30 | (0.15;0.44) | 1.00 | 1.01 |
| *λ_1_* | 13.06 | (8.68;15.76) | 1.00 | 1.00 |
| *λ_2_* | 13.52 | (9.18;17.91) | 1.00 | 1.01 |
| *λ_3_* | 13.11 | (8.61;15.80) | 1.00 | 1.00 |
| *λ_4_* | 10.20 | (7.35;11.95) | 1.00 | 1.00 |
| *λ_5_* | 14.26 | (12.18;17.14) | 1.00 | 1.00 |
| *λ_6_* | 14.31 | (12.13;17.15) | 1.00 | 1.00 |
| *λ_7_* | 14.17 | (10.86;17.07) | 1.00 | 1.01 |
| *λ_8_* | 14.15 | (10.92;17.00) | 1.00 | 1.00 |
| *λ_9_* | 14.21 | (10.94;17.00) | 1.00 | 1.00 |

*CI: Credibility interval
